# The STING in Non-Alcoholic Fatty Liver Diseases: Potential Therapeutic Targets in Inflammation-Carcinogenesis Pathway

**DOI:** 10.3390/ph15101241

**Published:** 2022-10-09

**Authors:** Juan Lv, Chunlei Xing, Yuhong Chen, Huihui Bian, Nanning Lv, Zhibin Wang, Mingming Liu, Li Su

**Affiliations:** 1 Institute of Translational Medicine, Shanghai University, Shanghai 200444, China; 2 School of Pharmacy, Bengbu Medical College, Bengbu 233030, China; 3 Lianyungang Second People’s Hospital, Lianyungang 222002, China; 4 Department of Critical Care Medicine, School of Anesthesiology, Naval Medical University, Shanghai 200020, China; 5 School of Pharmacy, Naval Medical University, Shanghai 200433, China

**Keywords:** non-alcoholic fatty liver disease, STING, inflammation, inhibitors, agonists

## Abstract

Non-alcoholic fatty liver disease (NAFLD), an important chronic disease, is one of the major causes of high mortality and creates a substantial financial burden worldwide. The various immune cells in the liver, including macrophages, NK cells, dendritic cells, and the neutrophils involved in the innate immune response, trigger inflammation after recognizing the damage signaled from infection or injured cells and tissues. The stimulator of interferon genes (STING) is a critical molecule that binds to the cyclic dinucleotides (CDNs) generated by the cyclic GMP-AMP synthase (cGAS) to initiate the innate immune response against infection. Previous studies have demonstrated that the cGAS-STING pathway plays a critical role in inflammatory, auto-immune, and anti-viral immune responses. Recently, studies have focused on the role of STING in liver diseases, the results implying that alterations in its activity may be involved in the pathogenesis of liver disorders. Here, we summarize the function of STING in the development of NAFLD and present the current inhibitors and agonists targeting STING.

## 1. Introduction

Non-alcoholic fatty liver disease (NAFLD), a disease characterized by hepatic steatosis, ranging from simple steatosis (non-alcoholic fatty liver, NAFL) to a more developed inflammatory and fibrogenic phase (non-alcoholic steatosis hepatitis, NASH) and even progress to hepatocellular carcinoma (HCC), causes enormous health problems and has created a financial burden worldwide [1,2,3]. Currently, NAFLD has become the second indication for liver transplantation in Western countries. NAFL-associated end-stage liver disease may also become one of the major indications of liver transplantation in China in the future. The annual health costs that are directly attributable to NAFLD are expected to exceed EUR 35 billion in four European countries (France, the UK, Germany, and Italy) and more than USD 100 billion in the United States [4]. NAFLD affects approximately 25% of the global population, with the highest prevalence in the Middle East (32%) and South America (31%), followed by Asia (27%), the US (24%), and Europe (23%) [5]. Hepatic inflammation is the main cause of liver injury, including NASH and its related diseases [6,7]. The pathogenesis of NASH is complex, and its occurrence and progression are regulated by hepatic lipid deposition, oxidative stress, insulin resistance, immune dysfunction, and apoptosis [8,9,10,11]. Emerging studies suggest that NAFLD-associated events significantly increase cancer mortality, cardiometabolic disease, and liver disease. The magnitude of these risks is particularly high when the disease progresses to NASH [12,13,14]. Moreover, based on its remarkable prevalence and persistent progressive features, there is no doubt that the burden of NAFLD will continue to increase in the absence of an effective control [15]. Unfortunately, even though its pathogenesis and potential therapeutic targets have been extensively reported, no drugs have yet been approved by the US Food and Drug Administration (FDA) for clinical use.

Factors influencing the development of NAFL/NASH are gradually being identified. Recently, the potential role of the stimulator of interferon genes (STING) and its downstream signaling pathways in the progression of NAFLD has been demonstrated [16,17,18]. STING, a pattern recognition receptor mainly residing on the endoplasmic reticulum (ER), requires palmitoylation at the Golgi apparatus to accomplish its activation after recognizing exogenous or self-DNA [19]. Subsequently, its downstream pathways including tank-binding kinase 1 (TBK1), interferon regulating factor (IRF)3, and nuclear factor kappa-B (NF-κB) are activated to induce the production of type Ⅰ interferon (IFN) and pro-inflammatory factors to regulate innate immune cell responses [20,21]. STING expression was found to be significantly upregulated in the liver of patients with NAFL [22], while STING expression in macrophages/Kupffer cells (KCs) could promote the progression of NAFLD via the mediation of hepatic inflammation and fibrosis [23,24], suggesting that STING activation promotes NAFLD progression. Long-term activation of the STING pathway in the liver aggravates inflammation, hepatocyte death, and compensatory proliferation, thereby promoting the inflammation-driven carcinogenesis pathway, suggesting that the inhibition of STING appears to be a potential strategy to slow the progression of inflammatory NASH. In contrast, some cancer cells were found to evade tumor immunity by down-regulating STING expression, suggesting that the inhibition of STING may also play a tumor-promoting role in established tumors. Therefore, the activation of cGAS/STING signaling may contribute to anti-tumor immunity in the treatment of HCC [25,26,27,28]. In summary, STING-targeted regulators are expected to be used for the treatment of NASH and NASH-associated HCC, respectively.

Currently, negative regulation of the STING signaling pathway is mainly carried out through post-translational modification, the regulation of protein interaction, and the promotion of protein degradation. Haag et al. [29] reported that compounds such as H-151 and C171 inhibit the palmitoylation modifications required for STING activation through covalent binding, while Li et al. demonstrated that Astin C, tetrahydro-γ-carboline derivatives, and SN-011 bind to STING mainly through the competitive inhibition of endogenous cyclic dinucleotides (CDNs) [30]. In addition, Palbociclib, a 4/6 inhibitor of cyclin-dependent kinases (CDKs), was recently found to directly target the Y167 site of STING and inhibit the activation of STING by blocking STING dimerization [31]. However, no studies have reported inhibitors that target STING for the treatment of NAFLD. Besides, studies on STING agonists targeting advanced solid tumors are still in clinical phase trials. Therefore, it is important to further clarify the role of STING and its downstream signaling pathway in NAFL/NASH and to find inhibitors and agonists for the treatment of NAFLD and NASH-related HCC in the future. Here, we summarize and discuss the role of STING in NAFLD, and also present the existing inhibitors and agonists targeting STING.

## 2. Structure and Signaling Pathway of STING

**Figure 1:**
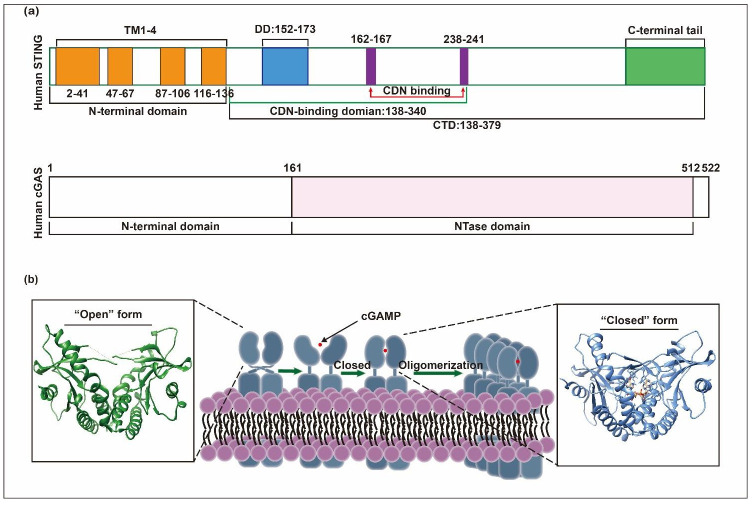
Structural basis of cGAS-STING [21,33]. (**a**) The domain composition of human-STING and human-cGAS. (**b**) STING oligomerization occurs after binding to cGAMP. Human-STING (colored in green, PDB ID:4EMU) and human-STING binds with 2′3′cGAMP (colored in cornflower blue, PDB ID:4LOH).

STING, encoded by TMEM173 genes, was firstly reported as an endoplasmic reticulum adaptor facilitating innate immune signaling in 2008 [32]. The structure and function of STING are highly conserved across species. Human STING contains 379 amino acids, while mouse STING encodes 378 amino acids. It consists of an N-terminal containing four transmembrane domains (TM1~4) and a C-terminal domain (CTD), mainly including the dimerization domain (DD) and the carboxyl-terminal tail (CTT). The N-terminal domain is mainly involved in anchoring STING to the ER or to other membrane structures. The DD, a highly conserved region, plays a critical role in the migration of STING to the perinuclear region and activating the downstream signaling pathways. The CTT is mainly responsible for the recruitment and activation of TBK1 and IRF3. In addition, the CTD domain contains two CDN-binding domains (CBD) that bind to cGAMP to activate STING. In its inactive state, STING is anchored to the ER as a butterfly-like dimer through several transmembrane domains (Figure 1). Once the cGAMP is bound, STING will undergo a conformational change that results in the tight binding of adjacent STING dimers to form oligomers that participate in the subsequent immune response.

**Figure 2:**
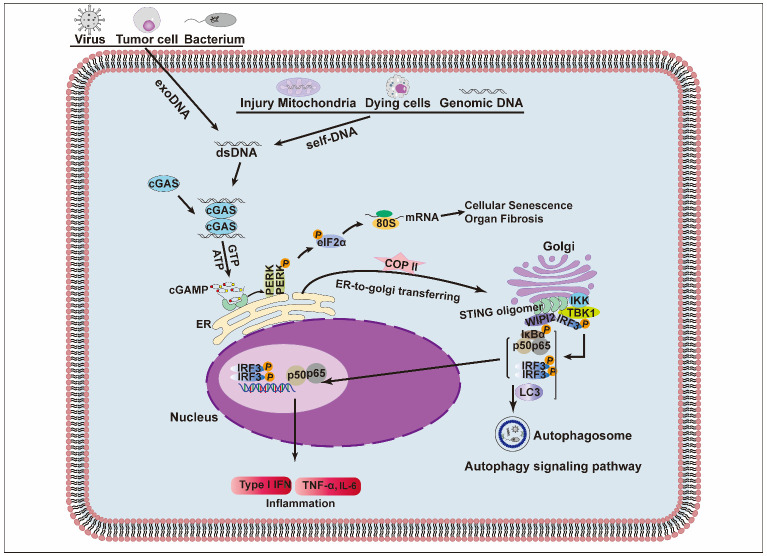
cGAS/STING signaling pathway in immunity [19,35,36]. Exogenous and self-DNA from viruses, tumors and dead cells, bacteria, damaged mitochondria, and genomes are recognized by cGAS, then by synthesized cGAMP from ATP and GTP. The binding of cGAMP to STING induces STING transfer from the ER to the Golgi apparatus and for self-oligomerization, which promotes the activation and translocation of NF-κB and IRF3 into the nucleus and exerts a pro-inflammatory effect by the generation of cytokines and type-I IFNs. It also induces LC3 to activate the autophagic signaling pathway, leading to cellular senescence and organ fibrosis via the STING-PERK-eIF2α pathway.

STING and its pathway have been found to play important roles in inflammation, autophagy, apoptosis, cellular senescence, anti-tumor immunity, and neurodegenerative diseases. The cGAS recognizes aberrant DNA derived from the cytoplasm and changes conformation to catalyze the production of cGAMP. Then, cGAMP binds and activates the STING located on the ER, causing STING to undergo a conformational change and transfer to the Golgi apparatus. Next, STING directly connects to sulfated glycosaminoglycans (sGAGs), initiating STING multimerization. Subsequently, the sequence motif named PLPLRT/SD, found at the C-terminal tail of STING, mediates the recruitment and activation of TBK1 and stimulates the phosphorylation of IRF3. Phosphorylated IRF3 dimerizes and translocates to the nucleus, triggering the expression of the type-I IFN gene [20,34]. In addition, STING also activates the NF-κB signaling pathway to generate the associated cytokines. Gui et al. also reported a novel mechanism whereby STING activates autophagy by inducing LC3 lipidation, which depends on WIPI2 and ATG5 rather than on TBK1 activation and IFN induction [35]. Furthermore, STING regulates the downstream PERK-elF2α pathway, leading to phosphorylation of the elF2α S51 site and the suppression of cap-dependent mRNA translation, to accelerate cellular senescence and lung and kidney fibrosis [36]. Activation of the above-mentioned signaling pathways contributes to the production of pro-inflammatory cytokines and amplifies inflammation (Figure 2). Although impermanent inflammation is essential to initiate the body’s defense responses against pathogen invasion, persistent or chronic inflammation can cause arthritis, cardiovascular diseases, autoimmune diseases, neurological diseases, and even cancer [37,38], indicating that restraining STING activation may provide a promising therapeutic strategy for inflammatory diseases. In contrast, appropriate activation of the cGAS-STING pathway can exhibit potential therapeutic effects in some cancers, and STING agonists can enhance anti-tumor activity. However, sustained auto-activation will induce chronic inflammation and ultimately promote tumor growth and metastasis [27,28,39,40]. Therefore, appropriate regulation of the cGAS-STING is particularly necessary.

## 3. NAFL/NASH and STING

NAFL is a type of simple steatosis characterized by hepatic steatosis without substantial inflammation or fibrosis [41]. Hepatic steatosis is defined as the deposition of large amounts of triglycerides in hepatocytes. Long-term fatty liver issues can cause the necrosis of hepatocytes, which may evolve into NASH, liver cirrhosis, or HCC [42,43,44].

The liver is an important immunological organ involved in the immune response; it possesses resident cells, including KCs, dendritic cells (DCs), hepatic stellate cells (HSCs), liver sinusoidal endothelial cells (LSECs), and blood circulating cells, such as natural killer cells (NK), monocytes, and neutrophils [45,46,47,48]. HSCs that normally remain quiescent transform into myofibroblasts with proliferative and fibrogenic characteristics upon recognition of injury or stimuli, and then secrete TGF-β and endothelin (ET)-1 to produce an abundant extracellular matrix (ECM), which together lead to liver fibrosis [49,50,51]. LSECs have a unique pore structure with loose intercellular connections and are highly permeable to substance exchange, due to the lack of basement membranes [52]. Liver injury or other pathological conditions disrupt the homeostasis of LSECs, causing capillarization and dysfunction and further activating KCs and HSCs, which plays a crucial role in steatosis, inflammation, and fibrosis and promotes the development of NAFLD [53,54,55,56]. It has been reported that NASH patients have an abnormal liver mitochondrial function and significantly higher cytoplasmic mtDNA content than healthy individuals [57]. When the Kupffer cells engulf apoptotic or dead hepatocytes, their self-DNA enters the cytoplasm and then activates cGAS–STING [58]. The activated KCs secrete cytokines such as TGF-β, TNF-α, and IL-1β, which activate HSCs and promote the proliferation of HSCs to drive liver fibrosis [59]. In addition, activated HSCs secrete large amounts of collagen, pro-inflammatory cytokines, chemokines, and inflammasome, which amplify inflammation and hepatic fibrosis [60]. Numerous studies have shown that these cells may be involved in the cGAS-STING signaling pathway to regulate the innate immune response [61,62].

The formation of p62 inclusions in hepatocytes is a key marker to distinguish simple fatty liver disease from NASH and predicts a poor prognostic outcome for subsequent liver carcinogenesis. The lipotoxic activation of TBK1 and p62 phosphorylation are critical steps in the accumulation of protein inclusion in hepatocytes. In the phase of NAFL, hepatic steatosis often provokes lipotoxic injuries to hepatocytes. A previous study showed that cGAS and STING, as upstream regulators, were involved in the lipotoxic activation of TBK1 and subsequent p62 phosphorylation in hepatocytes, leading to the formation of ubiquitin-p62 aggregates. The inclusions in hepatocytes promoted the development of steatohepatitis and liver cancer [63]. The evidence also demonstrates that STING activation promotes fat deposition by regulating lipid metabolism [22]. A study in *Drosophila* showed that the STING protein regulates lipid metabolism [64]. Furthermore, p62 was reported to mediate the autophagy pathway without differentiation between NASH and normal tissues. In contrast, the high expression of p62 in HCC suggests an impairment of autophagic flux and promotes tumor cell migration.

**Figure 3:**
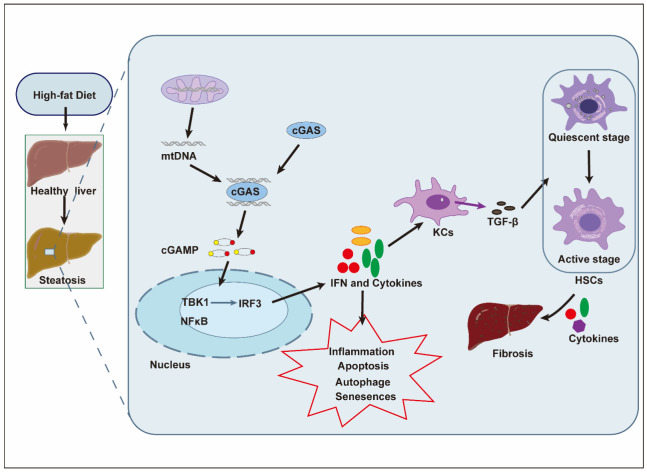
Role of the cGAS-STING pathway in HFD-induced NAFLD [22]. HFD-induced NAFLD manifests as steatosis, which leads to mitochondrial stress injury and the release of mitochondrial DNA (mtDNA) into the cytosol. Subsequently, the mtDNA is recognized by cGAS and produces cGAMP to activate the STING downstream pathways, including STING-TBK1-IRF3 and STING-NF-κB, which trigger the transcription of type-I IFNs and the production of pro-inflammatory cytokines, resulting in the hepatic inflammatory response. In addition, pro-inflammatory cytokines activate the function of macrophages/KCs and produce TGF-β, which activates HSCs and aggravates liver fibrosis in NASH.

The expression of STING is also upregulated in NAFLD. Previous studies showed that the STING-positive cells in the hepatic tissues of patients with NAFLD were mainly KCs/monocyte-derived macrophages and endothelial cells but not hepatocytes [22,24]. Moreover, the phosphorylation levels of TBK1 and IRF3 were observably up-regulated in mouse liver tissues after a high-fat diet (HFD) [22]. The pro-inflammatory effect of macrophages was enhanced by STING activation [23], producing IFNs, αSMA, TGF-β, and collagen A1, which strengthened fat deposition in hepatocytes and promoted HSC activation (Figure 3). In addition, the phosphorylation of STING downstream signals, such as TBK1, IRF3, JNK, and NF-κB was significantly increased in NAFLD, which induced more severe inflammation and fibrosis in the liver. In contrast, HFD-induced NAFLD was restrained in STING-deficient mice [17,22,65]. Another report also demonstrated that the lack of STING ameliorated NAFLD and reformed the gut bacterial community [66]. These pieces of evidence suggest that the activation of STING is indeed responsible for the development of NAFL/NASH. However, there are concerns regarding the translation from rodent findings to humans, which requires further investigation [67].

## 4. NASH-Associated HCC and STING

NASH is the primary risk factor for HCC, with approximately 10%–20% of patients developing cirrhosis and its potentially evolving to HCC within 5–10 years [68,69]. Currently, inflammation, oxidative stress, ER stress, and metabolic disorders have been reported as the pathogenesis of NASH-related HCC [70,71,72,73,74]. Among them, chronic liver inflammation is a mark of HCC, which evolution induces cell death and leads to the compensatory proliferation of hepatocytes [75]. Therefore, inflammation suppression may be beneficial in delaying disease progression. In hepatocellular carcinoma, studies show that two contradictory effects exist in STING activation-induced anti-tumor responses. Some studies have shown that STING activation mitigated HCC by enhancing anti-tumor immunity [76]. Once STING is activated in DCs and macrophages, cytokines are produced to initiate the innate immune effects directly and mediate adaptive immunity through the recruitment and activation of T cells [77]. During carcinogenesis, chromosomal instability (CIN), a persistent genomic change, including the expansion or deletion of chromosome copy number or structure, leading to micronucleus rupture and the leakage of genomic DNA into the cytoplasm, exacerbates tumor evolution [78,79,80,81]. As a cytosolic DNA sensor, cGAS links CIN to innate immunity by recognizing ruptured micronuclei and leads to the activation of STING and downstream NF-κB signaling, which suppresses cancer cell proliferation via producing IFNs, pro-inflammatory factors such as IL-6 and TNF-α, and chemokines, including CCL2, CCL5, and CXCL10 [82,83]. In addition, these cytokines or chemokines recruit NKs and DCs around the cancer tissues, to form a suppressive microenvironment that is responsible for the pro-defense role in tumor immunity. In addition, the clearance of tumor DNA also occurs as a consequence of STING activation, through non-immune functions such as autophagy, apoptosis, and necrosis [84]. In the mutagen-induced HCC model, the activation of STING allows cross-talking between hepatocytes and immune cells such as KCs. Further study showed that the deletion of STING accelerated HCC progression, whereas the application of a STING agonist resulted in suppressed tumor activity and increased T cell infiltration [76]. However, the literature also showed that aberrant activation of the STING pathway led to weakened immunity and promoted oncogenesis and metastasis [85]. The STING–TBK1–IRF3 pathway stimulated the production of immune checkpoint molecules, such as IFNβ, cytotoxic T-lymphocyte-associated protein 4 (CTLA-4), and programmed cell death ligand 1 (PD-L1), and inhibited T-cell activation, resulting in immune evasion [86].

Other reports showed that the hypoxic microenvironment within HCC inhibited the expression of cGAS. Therefore, cGAS-mediated DNA recognition and STING-TBK1-IRF3 pathway activation were inhibited [87,88]. Moreover, some tumors can evade the immune system by suppressing STING. For example, the MYC oncogene in triple-negative breast cancer inhibits STING expression by directly binding to the STING1 enhancer region, leading to tumor immune evasion [89]. Indeed, low levels of STING were found to be associated with poor prognosis in HCC patients [90]. Taken together, these studies indicate that STING agonists may have therapeutic values for HCC as monotherapy or/and adjuvants, in combination with immune checkpoint inhibitors.

## 5. STING Inhibitors and Agonists

As a pattern recognition receptor, STING recognizes foreign and self-DNA and changes its conformation to accomplish its activation and mediate the immune response. However, excessive STING activation is associated with inflammatory diseases. Meanwhile, the activation of STING shows surprising potential in the immunotherapy of tumors. Therefore, the development of inhibitors and agonists that modulate STING activity holds great promise for clinical treatment.

### 5.1. Covalent Inhibitors

The palmitoylation of STING in the Golgi apparatus is necessary for its activation [19]. Based on the previous study and a chemical screen, numerous compounds including C176, C178, and H151 have been identified. These compounds suppress palmitoylation modifications through covalent binding, which blocks the formation of STING polymers to regulate the STING signaling pathways [29]. H151 remarkably decreased the STING and IFN levels and systemic inflammation in pristane-induced lupus (PIL) mice, but clinical trials have not been conducted.

### 5.2. Noncovalent Inhibitors

Since covalent bonds are more powerful than non-covalent interactions, such as hydrogen bonds and salt bridges, typical covalent inhibitors are considered to have potential off-target effects and toxicity. Consequently, exploring reversible covalent or non-covalent inhibitors is essential. Astin C, isolated from *Aster tataricus,* is thought to inhibit STING via the specific binding between it and the C-terminal domain of STING. In addition, astin C breaks the recruitment of IRF3 onto the STING signalosome and suppresses the expression of IFN-β mediated by natural variations, including STING R232- and STING H232- [30]. Furthermore, Hong et al. [91] reported that SN-011 competes with CDN for the binding pocket of the STING dimer, thereby blocking CDN binding and activating STING. SN-011 is a potent and selective inhibitor of STING signal transduction, with an IC50 value of 76 nM. Elsewhere, palbociclib, an inhibitor of cyclin-dependent kinases (CDKs) 4/6, directly targets the STING Y167 and intercepts STING dimerization to restrain its activation. In dextran sulfate sodium salt-induced colitis and Trex1−/−-mediated autoinflammatory diseases, Palbociclib reduces STING-mediated inflammation and tissue injury. In addition, palbociclib can also block the formation of STING-TBK1 at the G166 locus [31]. Other commonly used STING inhibitors are shown in Table 1. Although several noncovalent STING inhibitors have been identified, none of them have been approved for clinical trials.

### 5.3. CDNs Agonists

CDNs agonists include natural agonists containing 2′3′-cGAMP, c-di-AMP, c-di-GMP, and 3′3′-cGAMP, along with synthetic agonists such as 2′3′-cGAMP (PS)2 (Rp/Sp), 2′3′-c-di-AM(PS)2 (Rp, Rp), cAIMP, cAIM (PS)2 Difluor (Rp/Sp), and other ramifications [95,96,97,98]. Native CDNs originate from mammalian cells or bacteria; they perform immune actions and physiological functions by activating STING and mediating the TBK1-IRF3-dependent pathway to produce type-I IFN [99,100]. In particular, 2′3′-cGAMP, as a second messenger generated by cGAS, has a high affinity and potently induces the production of IFNs [101,102]. For bacterial CDNs, the binding capacity of c-di-GMP and STING was stronger than that of c-di-AMP. Although native CDNs are essential for immunological study, their instability, negative charges, and hydrophilicity properties have limited their application. Therefore, structural modifications targeting CDNs are ongoing and have been shown to have an important influence on their efficacy and safety. Previous studies have shown that the CDNs of thiophosphate, rather than parent phosphoric acid CDNs, have higher bioactivity and a stronger activation of STING. MK-1454 is a CDNs of thiophosphate with efficient STING agonistic activity obtained based on P(III) chemical synthesis and structure optimization [103,104]. The phase-II clinical trials of MK-1454 applied to advanced solid tumors or lymphomas have been completed, showing inspiring effects when combined with pembrolizumab (a PD-1 antibody). A phase-I clinical trial (NCT04220866) of MK-1454 in combination with pembrolizumab for the treatment of head and neck squamous cell carcinoma (HNSCC) is currently underway. In addition, clinical trials have been allowed for MK-2118 from Merck, BMS-986301 from Bristol-Myers Squibb, SB 11285 from Spring Bank Pharmaceuticals, and other CDN-derived STING agonists shown in Table 1.

### 5.4. Non-CDN Agonists

Compared to the CDNs, the non-nucleotide STING agonists are more readily available in industrial production and application. Currently, widely used non-nucleotide agonists are mainly flavonoids, such as 5,6-dimethylxanthenone-4-acetic acid (DMXAA), flavone acetic acid (FAA), α-Mangostin, 10-carboxymethyl-9-acridanonecell (CMA) and dimeric amidobenzimidazole (diABZI) [105,106,107]. DMXAA, FAA, and CMA are only used for non-human cells in vitro studies, as they cannot bind and activate human STING [108]. Moreover, an oral non-nucleotide STING agonist named MSA-2 has been reported, which binds STING as a non-covalent dimer to generate a closed conformation. Studies have shown that MSA-2 eliminates tumors in colorectal cancer mice and exhibits good antitumor activity in combination with PD-1 antibody [109,110]. However, MSA-2 has not yet been studied in clinical trials.

SR-001 was identified as a STING agonist, based on a cellular activity screen and protein thermal shift assay. SR-001 is a pro-drug that functions via rapid conversion to SR-012 in cells. Given the instability of SR-001 and the low cellular permeability of SR-012, Wang et al. [111] identified SR-717 and found it to have cellular activity equivalent to that of SR-001. Interestingly, X-ray diffraction crystal analysis showed that SR-717 activated STING in the same manner as cGAMP and induced similar conformational changes in STING. In an invasive malignant melanoma mouse model, SR-717 suppressed tumor growth, blocked tumor metastasis, and promoted CD8+ T cell and NK cell recruitment around tumors to enhance anti-tumor immune responses [111,112]. Another STING agonist named PC7A is a PH-sensitive polymer, possessing a seven-membered ring with a tertiary amine [113]. The novel polyvalent STING agonist activates innate immunity and induces the prolonged generation of pro-inflammatory cytokines, such as IL-6 and IL-1β, via forming STING-PC7A condensates. Furthermore, there are many other STING agonists that are currently in clinical trials, some of which have potential therapeutic effects, as shown in Table 2.

## 6. Conclusions and Future Perspectives

The main characteristic of the NAFL phase is hepatic fat accumulation without inflammation, leading to hepatic steatosis and lipotoxic injury to hepatocytes. At this stage, STING is involved in the activation of TBK1 and p62 phosphorylation, which promotes the formation of large protein inclusions in hepatocytes [63]. As the disease progresses to NASH, the pathological features are inflammation, steatosis, hepatocyte injury, and various degrees of fibrosis [118]. cGAS recognizes the aberrant DNA and activates STING to trigger powerful immune responses that participate in the regulation of lipid metabolism and inflammatory responses, resulting in hepatic fat deposition and hepatocyte injury [22]. Thus, the inhibition of STING is a potential treatment for NAFLD/NASH. However, during HCC progression, STING activation has two contradictory effects: the enhancement of anti-tumor immune response by exerting adaptive immunity, the recruitment and activation of T cells [76], and the promotion of oncogenesis and metastasis by an exaggerated inflammatory response and the induction of PD-L1 expression [119]. Although STING agonists aiming at HCC are currently not available in clinics, some preclinical reports have shown that STING activation enhances anti-tumor responses and effectively reduces tumor size in the mouse models of mutagen-induced HCC [76], suggesting STING agonists as a potential treatment for HCC. Therefore, the modulation of STING and STING-mediated signaling pathways may delay the occurrence and development of NAFL to NASH and even to HCC. Due to the various forms of pathogenesis in the progression of NASH, research attention also should be focused on whether the accompanying side effects influence the expected treatments when STING or its pathway is activated or inhibited. Moreover, although temporary STING activation shows anti-viral and anti-tumor activity, persistent activation may promote cancer brought on by inflammation. Therefore, it is vital to perform more preclinical and clinical investigations to determine the safety, stability, and therapeutic efficacy of STING regulators.

To date, researchers have found or synthesized various inhibitors and agonists targeting STING via multiple screening methods or structural modifications. Some of these compounds have shown promising biological activities and have been tested in clinical trials. However, compounds targeting STING that are used to ameliorate NASH or reverse NASH-associated HCC have not been reported. Moreover, the off-target effects of covalent inhibitors and the species-specific problems found during screening compounds also limit the clinical studies of STING inhibitors. Consequently, further studies on the function of STING in the development of NASH and NASH-related HCC and exploring new STING regulators are particularly necessary.

## Figures and Tables

**Table 1:** Commonly used STING inhibitors.

Compound	Mechanism	Reference
Tetrahydroisoquinoline derivatives (Compound 18)	Target CBD binding pocket	[92]
Astin C	Inhibits STING-IRF3 interaction via the binding of the C-terminal domain of STING	[30]
SN-011	Target cyclic dinucleotide binding pocket	[91]
NO_2_-FAs	Suppresses STING palmitoylation	[93]
C-176, C-178	Suppresses murine STING palmitoylation	[29]
C170, C-171	Suppresses murine STING palmitoylation	
H-151	Suppresses human STING palmitoylation	
Gelsevirine	Targeting STING for K48 ubiquitination and degradation	[94]
Palbociclib	Directly targets STING Y167	[31]

**Table 2:** Part of the STING agonists currently under study.

Compound	Phase; Start Time; Notes	Reference/Clinical Trials ID
MK-1454	Ⅰ (2017): Intratumoral(IT); solid tumors or lymphomas	NCT03010176; NCT04220866; [103,104]
	Ⅱ (2020): IT; HNSCC	
MK-2118	Ⅰ (2017): IT/SQ; solid tumors or lymphomas	NCT03249792
BMS-986301	Ⅰ (2019): IV; advanced solid cancers	NCT03956680
BI-STING (BI1387446)	Ⅰ (2020): IT; advanced solid cancers	NCT04147234
SB 11285	Ⅰ (2019): IV; advanced solid tumors: melanoma, HNSCC, and solid tumor	NCT04096638
IMSA-101(GB492)	Ⅰ/Ⅱ (2019): IV; solid tumor	NCT04020185; CTR20211689
	Ⅰ (2021): I.V.; adult advanced malignant tumor	
E7766	Ⅰ (2020): IT; advanced solid tumors or lymphomas	NCT04144140; NCT04109092
	Withdrawn (Ⅰ(2020): urinary bladder neoplasms)	
MIW815(ADU-S100)	Terminated: Ⅰ (2016): advanced/metastatic solid tumors or lymphomas	NCT02675439; NCT03172936; NCT03937141
	Terminated: Ⅰ(2017): advanced/metastatic solid tumors or lymphomas)	
	Terminated: Ⅱ (2019: head and neck cancer)	
Compound 3	Preclinical: Support IV	[106]
a-Mangostin	Preclinical	[114]
GSK3745417	Ⅰ (2019): IV; advanced solid tumors	NCT03843359
BMS-986301	Ⅰ (2019): IT/IM; advanced solid cancers	NCT03956680
SYNB1891	Ⅰ (2019): IT; metastatic solid neoplasm lymphoma	NCT04167137
TAK-676	Ⅰ (2020): IV; advanced solid tumors	NCT04420884; NCT04879849; NCT04541108
	Early phase Ⅰ (2021): solid tumor	
SNX281	Ⅰ (2020): IV; solid tumors and lymphoma	NCT04609579
MSA-1	Preclinical	No data
MSA-2	Preclinical: orally available; antitumor	
SR-001	Preclinical: pro-drug	[111,112]
SR717	Preclinical: The compound was modified from the structure of SR-012 and was combined with cGAMP at the same position on STING; it could induce the same conformational change of STING and had a stronger response when combined with STING	
HG381	Ⅰ (2021): advanced solid tumors	NCT04998422; CTR20211765;
	Ⅰ (2021): advanced solid tumors Report: colorectal cancer; a better efficacy than ADB-S100	
KL340399	IND (2022: NMPA): IV; advanced solid tumor	CXHL2200223; CXHL2200224
NOX-66	Completed: Ⅰ/Ⅱ (2017): Cancer	NCT02941523; NCT04957290
	Ⅰ/Ⅱ (2021): metastatic castration-resistant prostate cancer and other solid tumors	
PC7A	Preclinical; form biomolecular condensates; intratumoral administration	[113,115,116]
IACS-8779	Preclinical (2021); IT; Canine glioblastoma	[117]
TAK-500	Recruiting:Ⅰ(2022), IV, HCC/Pancreatic cancer	NCT05070247

## Data Availability

Data sharing not applicable.
